# Ethyl 2-(3-ethyl­sulfinyl-5-methyl-1-benzo­furan-2-yl)acetate

**DOI:** 10.1107/S1600536809008460

**Published:** 2009-03-14

**Authors:** Hong Dae Choi, Pil Ja Seo, Byeng Wha Son, Uk Lee

**Affiliations:** a Department of Chemistry, Dongeui University, San 24 Kaya-dong Busanjin-gu, Busan 614-714, Republic of Korea; b Department of Chemistry, Pukyong National University, 599-1 Daeyeon 3-dong Nam-gu, Busan 608-737, Republic of Korea

## Abstract

The title compound, C_15_H_18_O_4_S, was prepared by the oxidation of ethyl 2-(3-ethyl­sulfanyl-5-methyl-1-benzofuran-2-yl)acetate with 3-chloro­peroxy­benzoic acid. The crystal structure is stabilized by aromatic π–π inter­actions between the benzene rings of neighbouring mol­ecules [centroid–centroid distance = 3.655 (3) Å] and by three inter­molecular C—H⋯O non-classical hydrogen bonds.

## Related literature

For the crystal structures of similar alkyl 2-(5-methyl-3-methyl­sulfinyl-1-benzofuran-2-yl)acetate derivatives, see: Choi *et al.* (2008*a* ▶,*b* ▶).
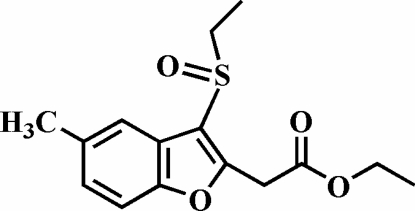


## Experimental

### 

#### Crystal data

C_15_H_18_O_4_S
*M* *_r_* = 294.36
Monoclinic, 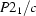

*a* = 7.9651 (5) Å
*b* = 17.397 (1) Å
*c* = 10.6902 (7) Å
β = 102.431 (1)°
*V* = 1446.60 (16) Å^3^
*Z* = 4
Mo *K*α radiation
μ = 0.23 mm^−1^
*T* = 293 K
0.40 × 0.35 × 0.30 mm

#### Data collection

Bruker SMART CCD diffractometer
Absorption correction: none
12350 measured reflections
3148 independent reflections
2949 reflections with *I* > 2σ(*I*)
*R*_int_ = 0.037

#### Refinement

*R*[*F* ^2^ > 2σ(*F* ^2^)] = 0.052
*wR*(*F* ^2^) = 0.114
*S* = 1.27
3148 reflections
184 parameters
H-atom parameters constrained
Δρ_max_ = 0.28 e Å^−3^
Δρ_min_ = −0.33 e Å^−3^

### 

Data collection: *SMART* (Bruker, 2001 ▶); cell refinement: *SAINT* (Bruker, 2001 ▶); data reduction: *SAINT*; program(s) used to solve structure: *SHELXS97* (Sheldrick, 2008 ▶); program(s) used to refine structure: *SHELXL97* (Sheldrick, 2008 ▶); molecular graphics: *ORTEP-3* (Farrugia, 1997 ▶) and *DIAMOND* (Brandenburg, 1998 ▶); software used to prepare material for publication: *SHELXL97*.

## Supplementary Material

Crystal structure: contains datablocks global, I. DOI: 10.1107/S1600536809008460/rk2133sup1.cif

Structure factors: contains datablocks I. DOI: 10.1107/S1600536809008460/rk2133Isup2.hkl

Additional supplementary materials:  crystallographic information; 3D view; checkCIF report

## Figures and Tables

**Table 1:** Hydrogen-bond geometry (Å, °)

*D*—H⋯*A*	*D*—H	H⋯*A*	*D*⋯*A*	*D*—H⋯*A*
C5—H5⋯O3^i^	0.93	2.64	3.507 (3)	155
C6—H6⋯O2^ii^	0.93	2.63	3.511 (3)	158
C9—H9*B*⋯O4^iii^	0.97	2.20	3.161 (3)	169

Symmetry codes: (i) 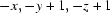; (ii) 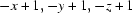; (iii) 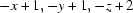.

**Fig. 1.**
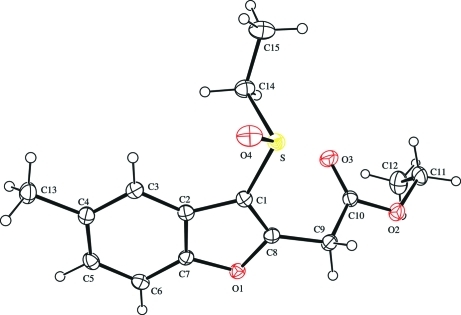
The molecular structure of the title compound with the atom numbering scheme. Displacement ellipsoids are drawn at the 50% probability level. H atoms are presented as a small spheres of arbitrary radius.

**Fig. 2.**
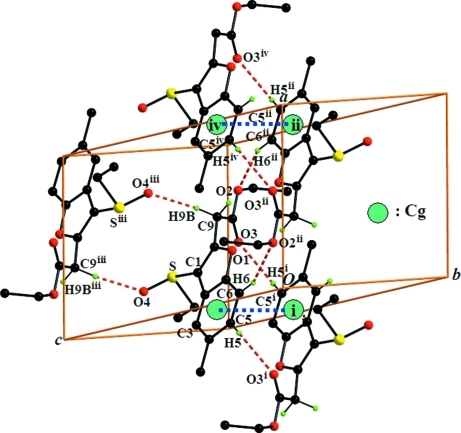
π-π and C–H···O interactions (dotted lines) in the title crystal structure. Cg denotes ring centroid. [Symmetry codes: (i) -x, -y+1, -z+1; (ii) -x+1, -y+1, -z+1; (iii) -x+1, -y+1, -z+2; (iv) x+1, y, z].

## supplementary crystallographic information

### Comment

As a part of our continuing studies on the synthesis and structure of alkyl 2-(5-methyl-3-methylsulfinyl-1-benzofuran-2-yl)acetate analogues, we have recently described the crystal structure of methyl 2-(5-methyl-3-methylsulfinyl-1-benzofuran-2-yl)acetate (Choi *et al.*, 2008*a*) and isopropyl 2-(5-methyl-3-methylsulfinyl-1-benzofuran-2-yl)acetate (Choi *et al.*, 2008*b*). Here we report the crystal structure of the title compound, ethyl 2-(3-ethylsulfinyl-5-methyl-1-benzofuran-2-yl)acetate (Fig. 1).

The benzofuran unit is essentially planar, with a mean deviation of 0.009 (2) Å from the least-squares plane defined by the nine constituent atoms. The molecular packing (Fig. 2) is stabilized by aromatic π-π interactions between the benzene rings of the adjacent molecules, with a *Cg*···*Cg*^i^ distance of 3.655 (3) Å (*Cg* is the centroid of the C2-C7 benzene ring; symmetry codes as in Fig. 2). The crystal packing is further stabilized by intermolecular C–H···O nonclassical hydrogen bonds; one between a benzene-H atom and the O atom of the C═O unit, a second between a benzene-H atom and the O atom of the ethoxy group, a third between an H atom of the methylene group bonded to carboxylate C atom and the S═O unit, respectively (Table 1 and Fig. 2; symmetry codes as in Fig. 2).

### Experimental

The 77% 3-chloroperoxybenzoic acid (247 mg, 1.1 mmol) was added in small portions to a stirred solution of ethyl 2-(3-ethylsulfanyl-5-methyl-1-benzofuran-2-yl)acetate (278 mg, 1.0 mmol) in dichloromethane (30 ml) at 273 K. After being stirred for 3 h at room temperature, the mixture was washed with saturated sodium bicarbonate solution and the organic layer was separated, dried over magnesium sulfate, filtered and concentrated in vacuum. The residue was purified by column chromatography (hexane-ethyl acetate, 1:2 *v*/*v*) to afford the title compound as a colourless solid [yield 80%, m.p. 381-382 K; Rf = 0.51 (hexane-ethyl acetate, 1:2 *v*/*v*)]. Single crystals suitable for *X*-ray diffraction were prepared by evaporation of a solution of the title compound in acetone at room temperature. Spectroscopic analysis: ^1^H NMR (CDCl_3_, 400 MHz) δ 1.27 (t, J = 6.96 Hz, 3H), 1.32 (t, J = 7.32 Hz, 3H), 2.45 (s, 3H), 3.31 (q, J = 7.32 Hz, 2H), 4.04 (s, 2H), 4.20 (q, J = 7.32 Hz, 2H), 7.17 (dd, J = 8.44 Hz and 1.48 Hz, 1H), 7.39 (d, J = 8.44 Hz, 1H), 7.66 (s, 1H); EI–MS 294 [*M*^+^].

### Refinement

All H atoms were geometrically positioned and refined using a riding model, with C–H = 0.93 Å for the aryl, 0.97 Å for the methylene, and 0.96 Å for the methyl H atoms. The *U*_iso_(H) = 1.2*U*_eq_(C) for the aryl and methylene H atoms, and 1.5*U*_eq_(C) for methyl H atoms.

### Crystal data

**Table:**

C_15_H_18_O_4_S	*F*(000) = 624
*M**_r_* = 294.36	*D*_x_ = 1.352 Mg m^−^^3^
Monoclinic, *P*2_1_/*c*	Mo *K*α radiation, λ = 0.71073 Å
Hall symbol: -P 2ybc	Cell parameters from 7772 reflections
*a* = 7.9651 (5) Å	θ = 2.3–28.2°
*b* = 17.397 (1) Å	µ = 0.23 mm^−^^1^
*c* = 10.6902 (7) Å	*T* = 293 K
β = 102.431 (1)°	Block, colourless
*V* = 1446.60 (16) Å^3^	0.40 × 0.35 × 0.30 mm
*Z* = 4	

### Data collection

**Table:**

Bruker SMART CCD diffractometer	2949 reflections with *I* > 2σ(*I*)
Radiation source: Fine-focus sealed tube	*R*_int_ = 0.037
graphite	θ_max_ = 27.0°, θ_min_ = 2.3°
Detector resolution: 10.0 pixels mm^-1^	*h* = −10→10
φ and ω scans	*k* = −22→22
12350 measured reflections	*l* = −13→13
3148 independent reflections	

### Refinement

**Table:**

Refinement on *F*^2^	Primary atom site location: Direct
Least-squares matrix: Full	Secondary atom site location: Difmap
*R*[*F*^2^ > 2σ(*F*^2^)] = 0.052	Hydrogen site location: Difmap
*wR*(*F*^2^) = 0.114	H-atom parameters constrained
*S* = 1.27	*w* = 1/[σ^2^(*F*_o_^2^) + (0.0313*P*)^2^ + 1.0305*P*] where *P* = (*F*_o_^2^ + 2*F*_c_^2^)/3
3148 reflections	(Δ/σ)_max_ < 0.001
184 parameters	Δρ_max_ = 0.28 e Å^−^^3^
0 restraints	Δρ_min_ = −0.33 e Å^−^^3^

### Special details

**Table:**

Geometry. All s.u.'s (except the s.u. in the dihedral angle between two l.s. planes) are estimated using the full covariance matrix. The cell s.u.'s are taken into account individually in the estimation of s.u.'s in distances, angles and torsion angles; correlations between s.u.'s in cell parameters are only used when they are defined by crystal symmetry. An approximate (isotropic) treatment of cell s.u.'s is used for estimating s.u.'s involving l.s. planes.
Refinement. Refinement of *F*^2^ against ALL reflections. The weighted *R*-factor *wR* and goodness of fit *S* are based on *F*^2^, conventional *R*-factors *R* are based on *F*, with *F* set to zero for negative *F*^2^. The threshold expression of *F*^2^ > σ(*F*^2^) is used only for calculating *R*-factors(gt) *etc*. and is not relevant to the choice of reflections for refinement. *R*-factors based on *F*^2^ are statistically about twice as large as those based on *F*, and *R*-factors based on ALL data will be even larger.

### Fractional atomic coordinates and isotropic or equivalent isotropic displacement parameters (Å^2^)

**Table:**

	*x*	*y*	*z*	*U*_iso_*/*U*_eq_	
S	0.28613 (7)	0.56219 (3)	0.92537 (5)	0.02864 (15)	
O1	0.34278 (18)	0.48031 (8)	0.59291 (13)	0.0264 (3)	
O2	0.69183 (19)	0.68219 (9)	0.71529 (15)	0.0346 (4)	
O3	0.4106 (2)	0.68573 (9)	0.71809 (18)	0.0420 (4)	
O4	0.2363 (2)	0.50522 (10)	1.01572 (15)	0.0407 (4)	
C1	0.2712 (3)	0.51603 (11)	0.77684 (19)	0.0251 (4)	
C2	0.1341 (3)	0.46731 (11)	0.70835 (19)	0.0243 (4)	
C3	−0.0237 (3)	0.44088 (11)	0.72840 (19)	0.0267 (4)	
H3	−0.0606	0.4539	0.8025	0.032*	
C4	−0.1244 (3)	0.39479 (11)	0.6356 (2)	0.0284 (4)	
C5	−0.0667 (3)	0.37595 (12)	0.5239 (2)	0.0303 (5)	
H5	−0.1357	0.3453	0.4623	0.036*	
C6	0.0892 (3)	0.40148 (12)	0.5024 (2)	0.0293 (4)	
H6	0.1266	0.3886	0.4285	0.035*	
C7	0.1861 (2)	0.44711 (11)	0.59661 (19)	0.0245 (4)	
C8	0.3903 (3)	0.52184 (11)	0.70459 (19)	0.0253 (4)	
C9	0.5568 (3)	0.56329 (12)	0.7224 (2)	0.0284 (4)	
H9A	0.6154	0.5470	0.6563	0.034*	
H9B	0.6280	0.5487	0.8045	0.034*	
C10	0.5396 (3)	0.64999 (12)	0.71761 (19)	0.0278 (4)	
C11	0.6973 (3)	0.76628 (13)	0.7098 (2)	0.0398 (6)	
H11A	0.8137	0.7838	0.7442	0.048*	
H11B	0.6235	0.7877	0.7623	0.048*	
C12	0.6400 (4)	0.79423 (16)	0.5758 (3)	0.0537 (7)	
H12A	0.7059	0.7693	0.5222	0.080*	
H12B	0.6567	0.8488	0.5734	0.080*	
H12C	0.5203	0.7826	0.5454	0.080*	
C13	−0.2954 (3)	0.36439 (14)	0.6536 (2)	0.0385 (5)	
H13A	−0.2898	0.3094	0.6617	0.058*	
H13B	−0.3839	0.3782	0.5810	0.058*	
H13C	−0.3213	0.3863	0.7298	0.058*	
C14	0.1037 (3)	0.62590 (13)	0.8750 (2)	0.0352 (5)	
H14A	0.1247	0.6597	0.8078	0.042*	
H14B	0.0016	0.5958	0.8406	0.042*	
C15	0.0745 (4)	0.67308 (15)	0.9860 (2)	0.0464 (6)	
H15A	0.0334	0.6405	1.0455	0.070*	
H15B	−0.0093	0.7122	0.9555	0.070*	
H15C	0.1806	0.6967	1.0281	0.070*	

### Atomic displacement parameters (Å^2^)

**Table:**

	*U*^11^	*U*^22^	*U*^33^	*U*^12^	*U*^13^	*U*^23^
S	0.0286 (3)	0.0315 (3)	0.0249 (3)	0.0021 (2)	0.00368 (19)	−0.0026 (2)
O1	0.0259 (7)	0.0274 (7)	0.0267 (7)	0.0001 (6)	0.0075 (6)	−0.0001 (6)
O2	0.0277 (8)	0.0286 (8)	0.0471 (9)	−0.0027 (6)	0.0068 (7)	0.0024 (7)
O3	0.0333 (9)	0.0303 (8)	0.0651 (12)	0.0018 (7)	0.0169 (8)	−0.0043 (8)
O4	0.0523 (10)	0.0428 (9)	0.0289 (8)	0.0086 (8)	0.0132 (7)	0.0077 (7)
C1	0.0267 (10)	0.0232 (10)	0.0251 (10)	0.0009 (8)	0.0047 (8)	0.0020 (8)
C2	0.0265 (10)	0.0199 (9)	0.0255 (10)	0.0029 (8)	0.0038 (8)	0.0044 (7)
C3	0.0286 (10)	0.0245 (10)	0.0281 (10)	0.0022 (8)	0.0084 (8)	0.0053 (8)
C4	0.0262 (10)	0.0218 (10)	0.0358 (11)	0.0017 (8)	0.0037 (8)	0.0066 (8)
C5	0.0316 (11)	0.0225 (10)	0.0331 (11)	0.0012 (8)	−0.0012 (8)	0.0003 (8)
C6	0.0347 (11)	0.0249 (10)	0.0277 (11)	0.0034 (9)	0.0056 (8)	−0.0003 (8)
C7	0.0230 (10)	0.0211 (9)	0.0294 (10)	0.0025 (8)	0.0055 (8)	0.0034 (8)
C8	0.0249 (10)	0.0228 (9)	0.0273 (10)	0.0033 (8)	0.0035 (8)	0.0030 (8)
C9	0.0244 (10)	0.0270 (10)	0.0340 (11)	0.0008 (8)	0.0072 (8)	0.0018 (8)
C10	0.0270 (10)	0.0297 (11)	0.0266 (10)	−0.0019 (8)	0.0055 (8)	−0.0016 (8)
C11	0.0368 (13)	0.0275 (11)	0.0533 (15)	−0.0086 (10)	0.0056 (11)	−0.0040 (10)
C12	0.0513 (16)	0.0412 (14)	0.0632 (18)	−0.0083 (12)	0.0006 (13)	0.0123 (13)
C13	0.0309 (12)	0.0327 (12)	0.0513 (14)	−0.0050 (9)	0.0078 (10)	0.0045 (10)
C14	0.0357 (12)	0.0345 (12)	0.0353 (12)	0.0064 (10)	0.0077 (9)	0.0016 (9)
C15	0.0588 (17)	0.0400 (14)	0.0425 (14)	0.0167 (12)	0.0150 (12)	0.0009 (11)

### Geometric parameters (Å, °)

**Table:**

S—O4	1.4960 (17)	C8—C9	1.485 (3)
S—C1	1.760 (2)	C9—C10	1.514 (3)
S—C14	1.815 (2)	C9—H9A	0.9700
O1—C8	1.377 (2)	C9—H9B	0.9700
O1—C7	1.384 (2)	C11—C12	1.488 (4)
O2—C10	1.341 (2)	C11—H11A	0.9700
O2—C11	1.465 (3)	C11—H11B	0.9700
O3—C10	1.202 (3)	C12—H12A	0.9600
C1—C8	1.350 (3)	C12—H12B	0.9600
C1—C2	1.450 (3)	C12—H12C	0.9600
C2—C7	1.391 (3)	C13—H13A	0.9600
C2—C3	1.398 (3)	C13—H13B	0.9600
C3—C4	1.388 (3)	C13—H13C	0.9600
C3—H3	0.9300	C14—C15	1.502 (3)
C4—C5	1.408 (3)	C14—H14A	0.9700
C4—C13	1.512 (3)	C14—H14B	0.9700
C5—C6	1.383 (3)	C15—H15A	0.9600
C5—H5	0.9300	C15—H15B	0.9600
C6—C7	1.380 (3)	C15—H15C	0.9600
C6—H6	0.9300		
			
O4—S—C1	107.72 (10)	H9A—C9—H9B	107.6
O4—S—C14	106.81 (10)	O3—C10—O2	124.1 (2)
C1—S—C14	96.70 (10)	O3—C10—C9	125.91 (19)
C8—O1—C7	105.99 (15)	O2—C10—C9	109.95 (17)
C10—O2—C11	116.92 (17)	O2—C11—C12	111.1 (2)
C8—C1—C2	107.49 (18)	O2—C11—H11A	109.4
C8—C1—S	124.25 (16)	C12—C11—H11A	109.4
C2—C1—S	128.26 (15)	O2—C11—H11B	109.4
C7—C2—C3	119.49 (19)	C12—C11—H11B	109.4
C7—C2—C1	104.49 (17)	H11A—C11—H11B	108.0
C3—C2—C1	136.00 (19)	C11—C12—H12A	109.5
C4—C3—C2	118.71 (19)	C11—C12—H12B	109.5
C4—C3—H3	120.6	H12A—C12—H12B	109.5
C2—C3—H3	120.6	C11—C12—H12C	109.5
C3—C4—C5	119.81 (19)	H12A—C12—H12C	109.5
C3—C4—C13	120.5 (2)	H12B—C12—H12C	109.5
C5—C4—C13	119.7 (2)	C4—C13—H13A	109.5
C6—C5—C4	122.3 (2)	C4—C13—H13B	109.5
C6—C5—H5	118.8	H13A—C13—H13B	109.5
C4—C5—H5	118.8	C4—C13—H13C	109.5
C7—C6—C5	116.40 (19)	H13A—C13—H13C	109.5
C7—C6—H6	121.8	H13B—C13—H13C	109.5
C5—C6—H6	121.8	C15—C14—S	110.46 (17)
C6—C7—O1	125.83 (18)	C15—C14—H14A	109.6
C6—C7—C2	123.28 (19)	S—C14—H14A	109.6
O1—C7—C2	110.86 (17)	C15—C14—H14B	109.6
C1—C8—O1	111.17 (17)	S—C14—H14B	109.6
C1—C8—C9	132.92 (19)	H14A—C14—H14B	108.1
O1—C8—C9	115.91 (17)	C14—C15—H15A	109.5
C8—C9—C10	114.04 (17)	C14—C15—H15B	109.5
C8—C9—H9A	108.7	H15A—C15—H15B	109.5
C10—C9—H9A	108.7	C14—C15—H15C	109.5
C8—C9—H9B	108.7	H15A—C15—H15C	109.5
C10—C9—H9B	108.7	H15B—C15—H15C	109.5
			
O4—S—C1—C8	−135.83 (18)	C3—C2—C7—C6	−0.1 (3)
C14—S—C1—C8	114.09 (19)	C1—C2—C7—C6	−178.86 (18)
O4—S—C1—C2	45.4 (2)	C3—C2—C7—O1	178.44 (17)
C14—S—C1—C2	−64.7 (2)	C1—C2—C7—O1	−0.3 (2)
C8—C1—C2—C7	0.3 (2)	C2—C1—C8—O1	−0.2 (2)
S—C1—C2—C7	179.27 (15)	S—C1—C8—O1	−179.20 (13)
C8—C1—C2—C3	−178.1 (2)	C2—C1—C8—C9	179.7 (2)
S—C1—C2—C3	0.8 (3)	S—C1—C8—C9	0.7 (3)
C7—C2—C3—C4	0.1 (3)	C7—O1—C8—C1	0.0 (2)
C1—C2—C3—C4	178.4 (2)	C7—O1—C8—C9	−179.93 (16)
C2—C3—C4—C5	−0.3 (3)	C1—C8—C9—C10	−67.8 (3)
C2—C3—C4—C13	179.55 (18)	O1—C8—C9—C10	112.1 (2)
C3—C4—C5—C6	0.3 (3)	C11—O2—C10—O3	−1.6 (3)
C13—C4—C5—C6	−179.5 (2)	C11—O2—C10—C9	179.64 (18)
C4—C5—C6—C7	−0.3 (3)	C8—C9—C10—O3	10.4 (3)
C5—C6—C7—O1	−178.14 (18)	C8—C9—C10—O2	−170.85 (17)
C5—C6—C7—C2	0.2 (3)	C10—O2—C11—C12	−83.1 (3)
C8—O1—C7—C6	178.71 (19)	O4—S—C14—C15	66.5 (2)
C8—O1—C7—C2	0.2 (2)	C1—S—C14—C15	177.38 (18)

### Hydrogen-bond geometry (Å, °)

**Table:**

*D*—H···*A*	*D*—H	H···*A*	*D*···*A*	*D*—H···*A*
C5—H5···O3^i^	0.93	2.64	3.507 (3)	155
C6—H6···O2^ii^	0.93	2.63	3.511 (3)	158
C9—H9B···O4^iii^	0.97	2.20	3.161 (3)	169

Symmetry codes: (i) −*x*, −*y*+1, −*z*+1; (ii) −*x*+1, −*y*+1, −*z*+1; (iii) −*x*+1, −*y*+1, −*z*+2.
